# Supplementary material for: Stability of African Swine Fever Virus in Soil and Options to Mitigate the Potential Transmission Risk
Source: Pathogens. 2020 Nov 23;9(11):977. doi: 10.3390/pathogens9110977 (PMC7700497; doi:10.3390/pathogens9110977)
Supplement: Supplementary file 1 [file pathogens-09-00977-s001.zip › pathogens-998682supp.pdf]

**Supplements:**

Table S1: Parameters of purchased potting soil

| Parameter | Commercial Potting Soil |
|---|---|
| **pH** | 5.8 |
| **Organic Material** | 65% |
| **Salt Content** | 2.0 mg/L |
| **Nitrogen** | 250 mg/L |
| **Phosphorus $P_2O_5$** | 250 mg/L |
| **Potassium Oxide $K_2O$** | 450 mg/L |
| **Magnesium** | 170 mg/L |
| **Sulfur** | 130 mg/L |

Table S2: Soil analysis results

| Parameter (%Total Mass) | Yard Soil | Swamp | Forest Soil 1 | Forest Soil 2 | Beach Sand |
|---|---|---|---|---|---|
| **pH** | **6.7** | **5.1** | **4.1** | **3.2** | **6.6** |
| Potassium | 0.046 | 0.025 | 0.053 | 0.034 | 0.017 |
| Magnesium | 0.086 | 0.092 | 0.053 | 0.04 | 0.057 |
| **Organic material** | **2.4** | **71.8** | **14.4** | **30.9** | **0.1** |
| Phosphorus | 0.094 | 0.088 | 0.037 | 0.050 | 0.016 |
| Nitrogen | 0.12 | 2.25 | 0.034 | 0.80 | N/A |
| Sulfur | 0.02 | 0.77 | 0.04 | 0.09 | 0.0044 |
